# Safety and effectiveness of specific oral tolerance induction to cow’s milk

**DOI:** 10.1186/2045-7022-5-S3-P156

**Published:** 2015-03-30

**Authors:** Elena Finelli, Joana Belo, Sara Prates, Miguel Paiva, Paula Leiria Pinto

**Affiliations:** 1 Hospital de Dona Estefania, Lisbon, Portugal

## Background and aim

Specific oral tolerance induction (SOTI) to cow/s milk (CM) can be an alternative for children who have not achieved tolerance spontaneously. The aim of this study was to assess safety and effectiveness of SOTI.

## Methods

Patients (n=25; median age 11.7 years) with IgE-mediated persistent cow/s milk allergy (CMA) were enrolled into a specific oral tolerance induction (SOTI) program, performed by offering progressively increasing amounts of pure pasteurized cow's milk (CM). Different protocols were used, with flexible increasing doses, suitable for the characteristics of each patient.

## Results

In 23 patients we started a standard cluster protocol using unheated CM (UCM) - oral ingestion of increasing doses, always in a hospital setting, until reaching the target dose of 200 mL/day. In 2 cases, SOTI was performed with baked milk (BM). Among 23 who started SOTI with UCM, 12 completed the protocol successfully, 7 are still ongoing, 2 were changed to BM and in 3 patients SOTI failed to improve the condition, for a variety of reasons, including unresponsive gastric pain (2) and *de novo* diagnosis of eosinophilic esophagitis (1). Of the 4 patients who started or were changed to BM, 2 ended the protocol with (complete or partial) success, one is still ongoing and the last one was not able to progress beyond 0.5 mL. In summary, SOTI were completed with success in 14 patients (56%) - 12 with UCM and 2 with BM, and failed to improve the condition in 4 cases - 3 with UCM and 1 with BM. In 7 patients the protocol is still ongoing - 6 with UCM and 1 with BM. There were adverse reactions in 80% of cases, 3 of which with severe reactions (anaphylaxis), always in the induction phase.

## Conclusions

In our experience, SOTI is a valid option for persistent or severe CMA, and an individualized dosing regimen is important, paying attention to adverse reactions and planning alternative strategies to overcome the difficulties with the increase in dose. Replace CM with baked milk could be a well accepted possibility for who react to unheated milk. It is still unclear if the tolerance status achieved by SOTI is permanent or if regular CM intake will be required indefinitely.

